# Pharyngeal Co-Infections with Monkeypox Virus and Group A *Streptococcus*, United States, 2022

**DOI:** 10.3201/eid2909.230469

**Published:** 2023-09

**Authors:** Robyn M. Kaiser, Shama Cash-Goldwasser, Nicholas Lehnertz, Jayne Griffith, Alison Ruprecht, John Stanton, Amanda Feldpausch, Jessica Pavlick, Charles A. Bruen, David Perez-Molinar, S. Rebecca Peglow, Omobosola O. Akinsete, Sapna Bamrah Morris, Elliot Raizes, Christopher Gregory, Ruth Lynfield

**Affiliations:** HealthPartners Regions Hospital, Saint Paul, Minnesota, USA (R.M. Kaiser, C.A. Bruen, D. Perez-Molinar, S.R. Peglow, O.O. Akinsete);; Centers for Disease Control and Prevention, Atlanta, Georgia, USA (S. Cash-Goldwasser, S. Bamrah Morris, E. Raizes, C. Gregory);; Minnesota Department of Health, Saint Paul (N. Lehnertz, J. Griffith, A. Ruprecht, R. Lynfield);; Positive Impact Health Centers, Decatur, Georgia, USA (J. Stanton);; Emory University, Atlanta (J. Stanton);; Georgia Department of Public Health, Atlanta (A. Feldpausch, J. Pavlick)

**Keywords:** mpox, monkeypox, monkeypox virus, *Streptococcus pyogenes*, pharynx, pharyngeal disease, co-infection, bacteria, streptococci, viruses, zoonoses, Group A *Streptococcus*

## Abstract

We report 2 cases of pharyngeal monkeypox virus and group A *Streptococcus* co-infection in the United States. No rash was observed when pharyngitis symptoms began. One patient required intubation before mpox was diagnosed. Healthcare providers should be aware of oropharyngeal mpox manifestations and possible co-infections; early treatment might prevent serious complications.

During the ongoing mpox outbreak that began in 2022, severe oropharyngeal manifestations of mpox have been described (*1*–*3*). Co-infections have been diagnosed frequently in patients with mpox, notably sexually transmitted infections (*1*,*2*). We report on 2 cases of co-infection with pharyngeal monkeypox virus (MPXV) and group A *Streptococcus* (GAS) in patients in the United States.

## The Study

In August 2022, the Centers for Disease Control and Prevention was consulted about 2 patients. Patient A had GAS pharyngitis, suspected mpox with pharyngeal manifestations, and airway compromise; patient B had confirmed mpox and pharyngitis and pharyngeal swab samples that tested positive for 3 pathogens, including MPXV.

**Figure 1:**
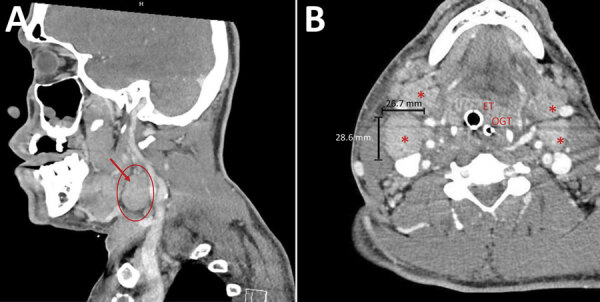
Computed tomography scans of patient’s neck in study of pharyngeal co-infections with monkeypox virus and group A *Streptococcus*, United States, 2022. A) Sagittal view of neck of patient A (39-year-old man) showing massive cervical lymphadenopathy (red circle and arrow). B) Axial view of hypopharynx of patient A after intubation with endotracheal tube. Asterisks show enlarged cervical lymph nodes. Patient was dependent on endotracheal tube because of soft tissue edema along the airway. ET, endotracheal tube; OGT, orogastric tube.

In August 2022, patient A, a 39-year-old man who had a history of substance use disorder and unstable housing, was seen at an emergency department because of severe odynophagia and myalgias. Physical examination revealed posterior oropharyngeal erythema, uvula edema, and tonsillar exudates. A pharyngeal swab sample was PCR positive for GAS; he received 1 dose of oral dexamethasone and was prescribed penicillin. The patient returned 4 days later because dysphagia, dyspnea, and a new maculopapular rash on his arms and chest had developed. He was treated with epinephrine, methylprednisolone, and intravenous dexamethasone for a presumed allergic reaction to penicillin. A computed tomography scan of his neck showed substantial cervical lymphadenopathy (Figure 1, panel A) and extensive soft tissue edema and inflammation of the soft palate, uvula, tonsils, epiglottis, and retropharyngeal tissues. Flexible laryngoscopy showed ulcerative, vesicular lesions on the epiglottis. He left the emergency department against medical advice with prescriptions for clindamycin and dexamethasone.

**Figure 2:**
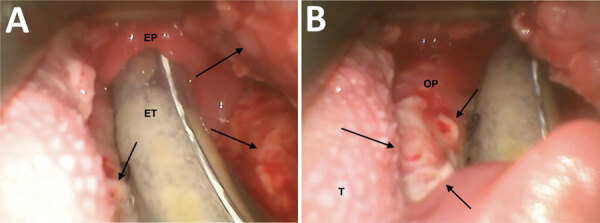
Video laryngoscopy images of patient larynx and pharynx in study of pharyngeal co-infections with monkeypox virus and group A *Streptococcus*, United States, 2022. A) View of oropharynx, hypopharynx, and laryngeal inlet of patient A (39-year-old man). Arrows indicate mpox lesions. B) Detailed view of mpox lesions. Arrows indicate several lesions. EP, epiglottis; ET, endotracheal tube; OP, oropharynx (lateral wall); T, tongue.

The next day, the patient was found lying on the ground, obtunded and with labored breathing, and was brought to the emergency department. He was immediately intubated and admitted to intensive care. A repeat computed tomography scan of his neck showed that his airway was dependent on the endotracheal tube; he had extensive soft tissue edema and cervical lymphadenopathy (Figure 1, panel B). He was treated for anaphylaxis with epinephrine and methylprednisolone and also received broad-spectrum antimicrobial drugs. He had a diffuse papulopustular rash consistent with mpox (*4*). Results of HIV antigen-antibody and PCR tests were negative. Video laryngoscopy showed edema and erythema of the pharynx, uvula, and epiglottis and multiple ulcers within the pharynx (Figure 2). PCR results were negative for herpes simplex and varicella zoster virus in skin lesion samples. Swab samples were collected from skin and pharyngeal lesions to test for orthopoxvirus (OPXV) by PCR. On hospitalization day 3, histological examination of a skin lesion punch biopsy was consistent with OPXV infection, and the patient was started on intravenous tecovirimat. On hospital day 4, PCR results of all swab samples from the skin (PCR cycle threshold 16.63 for left thigh and 17.02 for right neck) and pharyngeal (cycle threshold 17.30) lesions were positive for OPXV, and he was given intravenous cidofovir. He had negative test results for Epstein-Barr virus, cytomegalovirus, syphilis, and pharyngeal gonorrhea and chlamydia. Over the next several days, the patient’s skin lesions crusted, and airway edema decreased. He was extubated on hospital day 8. We obtained exposure history; the patient denied contact with persons who had mpox and said his last sexual encounter was with a female partner 4 weeks before symptom onset. He was discharged on hospitalization day 10.

In July 2022, patient B, a 36-year-old man with HIV infection (374 CD4+ cells/mm^3^; viral load was suppressed on antiretroviral treatment) sought care at a clinic for a genital rash. He had engaged in anal and oral sex with multiple male partners during the previous 30 days. A swab sample from the rash tested positive for OPXV by PCR. A swab sample from his pharynx tested positive for *Neisseria gonorrhoeae* by PCR, and a rapid plasma reagin test had a positive titer of 1:16 (titer was 1:2 in March 2022). The patient received intramuscular ceftriaxone and penicillin, and his rash resolved. He returned to the clinic 8 weeks later with severe odynophagia, but no rash was observed after examination. He had a gray-white exudate and ulcers in his pharynx from which swab samples were collected. He was empirically treated with 1 dose of ceftriaxone and a course of oral doxycycline. He returned 3 days later with substantial left-sided anterior cervical lymphadenopathy (>2 cm) and was prescribed oral penicillin, after which his symptoms improved. Results from oropharyngeal swab samples were positive for GAS, *N. gonorrhoeae*, and OPXV by PCR.

## Conclusions

We show that MPXV infections of the pharynx can co-occur with other oropharyngeal infections. Similar to findings from other reported cases in the literature, patient A illustrates that mpox manifestations can be oropharyngeal and include pharyngitis, odynophagia, epiglottitis, and oral and tonsillar lesions (*1*–*3*). In both of these cases, a rash was not noted at the time of pharyngeal symptoms. If a patient is suspected of having mpox-related oropharyngeal lesions, those lesions should be tested for OPXV/MPXV; if lesions exist at multiple sites, samples from all sites should be tested. Furthermore, healthcare providers should consider testing patients with suspected or confirmed mpox and pharyngeal symptoms for GAS, sexually transmitted infections, and other infections, guided by clinical findings and epidemiologic risk.

During the 2003 mpox outbreak in the United States, oropharyngeal lesions and considerable cervical and tonsillar lymphadenopathy developed in an otherwise healthy child with mpox who was hospitalized with dyspnea and dysphagia, but intubation was not required (*5*). During the ongoing outbreak, severe or critical illness secondary to oropharyngeal mpox manifestations has been described, albeit often in persons with advanced HIV disease (*3*,*6*). In 2 reported cases, patients with mpox required intubation secondary to airway compromise. In contrast to patient A in our report, those patients had underlying immunocompromising conditions (*3*). Healthcare providers should consider early antiviral treatment for patients with suspected or laboratory-confirmed mpox disease who have severe clinical manifestations (*7*), including oropharynx involvement, or have comorbidities that increase their risk for severe disease (*8*).

We were unable to determine the relative contribution of MPXV to illness compared with other pathogens in the 2 cases. Although GAS might have been a colonizing organism, GAS carriage among adults is uncommon (*9*). Both patients had clinical features and laboratory results consistent with GAS infection (*10*,*11*), for which antimicrobial drug treatment is recommended (*11*), and were treated accordingly. OPXV detection in samples from the oropharynx of patient B might have represented ongoing infection; the effect of mpox antiviral treatment on viral clearance is unknown.

Corticosteroids were used initially in patient A until mpox was suspected. Short courses of corticosteroids are used to treat severe acute pharyngitis symptoms (*12*) and pharyngeal edema (*13*). Corticosteroids can decrease duration and severity of symptoms in patients with GAS pharyngitis; however, given the potential adverse effects of steroids and effectiveness of antimicrobial drugs, systemic steroids are generally not recommended (*11*). Further studies are needed to determine whether corticosteroids have a role in mpox treatment, including in patients with complications such as pharyngeal edema or massive cervical lymphadenopathy.

In summary, healthcare providers should be aware that MPXV infections of the pharynx can be severe, can co-occur with other pharyngeal infections, and can manifest in the absence of a rash. Early antiviral treatment of mpox in patients with oropharyngeal manifestations and early diagnosis and treatment of pharyngeal co-infections might prevent serious complications.
